# The efficacy of negative pressure wound therapy after hepatopancreatobiliary surgery: A systematic review and meta-analysis

**DOI:** 10.12669/pjms.38.8.6601

**Published:** 2022

**Authors:** Bei Ren, XiaoJuan Jiang, Jin Chen, JunJun Mo

**Affiliations:** 1 Bei Ren, Department of Wound Stoma Outpatient Care, The First People’s Hospital of Wenling, Wenling, Zhejiang, China; 2 XiaoJuan Jiang Department of Endocrinology, The First People’s Hospital of Wenling, Wenling, Zhejiang, China; 3 Jin Chen, Outpatient Management Office, The First People’s Hospital of Wenling, Wenling, Zhejiang, China; 4 JunJun Mo, Department of PICC Outpatient, The First People’s Hospital of Wenling, Wenling, Zhejiang, China

**Keywords:** Surgical site infection, Seroma, hematoma, Negative pressure wound therapy, Morbidity, Hospital re-admission

## Abstract

**Objective:**

To evaluate the comparative influence of NPT and standard surgical dressing administration on incidence risk for surgical site infections, complications, and hospital re-admission after hepatopancreatobiliary surgery.

**Methods:**

Five databases were systematically searched according to PRISMA guidelines. These databases included Web of Science, MEDLINE, CENTRAL, EMBASE, and Scopus for eligible studies published prior to March 2021. With eligible studies, we conducted a random-effects meta-analysis to evaluate comparative outcomes such as superficial surgical infection, deep surgical infection, seroma incidence, hematoma incidence, and hospital re-admission in patients receiving NPT or standard surgical dressings after hepatopancreatobiliary surgery.

**Results:**

The search strategy yielded 963 studies, with six studies meeting inclusion criteria. Odds of superficial surgical site infection (OR: 1.58), deep surgical site infection (1.43), seroma complication (1.64), hematoma complication (0.40) were insignificantly different between patients receiving NPT and standard surgical dressing. The odds of hospital re-admission rate (2.37), however, were elevated in patients receiving standard surgical dressing relative to those receiving NPT.

**Conclusion:**

This meta-analysis shows that NPT usage slightly reduces risk of hospital readmission as compared to standard surgical dressing. We did not observe any significant effect of NPT on superficial, deep surgical infections, seroma, and haematoma outcomes following hepatopancreatobiliary surgery. These findings may aid clinicians in stratifying risk and selecting treatment strategy in patients undergoing hepatopancreatobiliary surgery.

## INTRODUCTION

Surgical site infection incidence for patients undergoing abdominal surgeries such as hepatopancreatobiliary surgery has been reported to be as high as 40%.^1^–^3^ Although medical advances have limited mortality in patients undergoing hepatopancreatobiliary surgery, recent studies indicate that morbidity remains high.^4^ Surgical site infections increase patient discomfort, delay adjuvant treatments, impose higher financial burdens, and lowers quality of life.^5^

Surgical site infection in hepatopancreatobiliary surgery patients typically occurs due to several pre-, intra-, and post-operative factors.^1^,^6^ Ceppa et al.^1^ reported discrepancies in surgical technique, execution, and wound management to be the most critical risk factor impacting wound soilage. They also noted that surgical site infections can develop either as superficial (i.e. skin and subcutaneous tissue), deep (i.e. fascia and muscle), or organ space infections.

Negative pressure wound therapy (NPT) has attracted much attention for facilitating wound recovery.^7^–^10^ NPT applies sub-atmospheric pressure on wound sites, leading to improved wound perfusion, granular tissue formation, exudate removal, and reduced microbial colonization.^11^–^13^ Specifically, studies suggest that the reverse-tissue expansion effect generated by NPT exploits the viscoelastic properties of the skin (i.e. the crinkle effect) to facilitate vascularity and mitotic activity at the wound site.^14^–^16^

Several studies have attempted to compare NPT and standard surgical dressing practices in terms of morbidity-related outcomes in patients undergoing hepatopancreatobiliary surgery.^7^–^10^,^17^,^18^ Nonetheless, no consensus currently exists in the literature regarding the impact of these different wound therapies on superficial and deep surgical site infections in hepatopancreatobiliary surgery patients. While some studies reported fewer superficial surgical site infections in patients receiving NPT,^8^,^10^,^17^ others noted either negligible differences or the opposite trend.^7^,^18^ Similarly, some studies have fewer deep surgical site infections in patients receiving NPT,^7^,^17^ while others have noted the opposite effect.^8^,^9^,^18^

To the best of our knowledge, no study has attempted to evaluate comparative morbidity when using NPT or standard surgical dressings after hepatopancreatobiliary surgery. We therefore sought to perform a meta-analysis of the available body of evidence on this subject. We attempted to evaluate the comparative impact of NPT and standard surgical dressing strategies on superficial surgical infection, deep surgical infection, seroma complication, hematoma complication, and hospital re-admission rates in patients undergoing hepatopancreatobiliary surgery. The present study aims to increase clinical awareness among surgeons concerning how wound therapy impacts patients undergoing hepatopancreatobiliary surgery.

## METHODS

This meta-analysis was conducted according to PRISMA (Preferred Reporting Items for Systematic Reviews and Meta-Analyses) guidelines.^19^

### Data search strategy:

We searched five scientific databases (Web of Science, MEDLINE, CENTRAL, EMBASE, and Scopus) for eligible studies published prior to March 2021. A number of MeSH keywords were used in combination, including “hepatopancreatobiliary”, “hepatic resection”, “pancreatic resection”, “negative pressure wound therapy”, “standard surgical dressing”, “surgical infection site, superficial”, “surgical infection site, deep”, “complications”, “seroma”, and “hematoma”. References cited by included studies were manually scanned to identify additional relevant studies. The inclusion criteria were as follows:

- Studies comparing superficial or deep surgical infection rates between patients receiving NPT and standard surgical dressing strategies after hepatopancreatobiliary surgery.
- Studies comparing post-surgical complications, including seroma, hematoma, and re-admission rates, between patients receiving NPT and standard surgical dressing strategies after hepatopancreatobiliary surgery.
- Studies involving human participants.
- Studies conducted as randomized controlled trials, controlled clinical trials, or cohort trials.
- Studies published in peer-reviewed scientific journals.
- Studies published in English.

Study screening was performed independently by two reviewers. Disagreements were resolved through discussions with a third reviewer.

### Quality assessment:

Bias risk within included randomized controlled trials was assessed using Cochrane’s risk of bias assessment tool (Sterne et al., 2016). Bias risk within included cohort trials was appraised using the ROBINS-I tool.^20^ Methodological quality appraisal was performed independently by two reviewers. Again, a third reviewer arbitrated any disputes.

### Data analysis:

A within-group meta-analysis was performed using Comprehensive Meta-analysis software version 2.0.^21^ The meta-analysis was conducted based on a random-effects model.^22^ We calculated odds ratios to evaluate the odds of superficial surgical infection, deep surgical infection, seroma complications, hematoma complications, and re-admission rates between patients receiving negative pressure wound therapy and standard surgical dressings after hepatopancreatobiliary surgery. I^2^ values were computed to assess heterogeneity (0-25%: negligible, 25%-75%: moderate, ≥75%: substantial.^23^ When studies provided medians and ranges, we used a previously established method by^24^ to convert these values into means and standard deviations. Publication bias was evaluated using Duval and Tweedy’s trim and fill procedure.^25^ The significance level for this study was determined to be 5%.

## RESULTS

**Fig.1:**
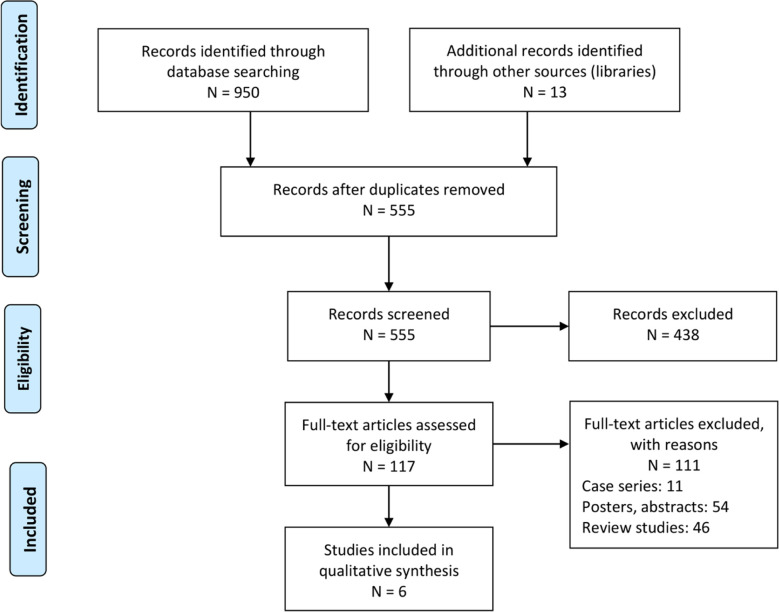
PRISMA flowchart detailing literature search and screening strategy.

Our literature search identified 950 studies for inclusion. Reference section screening yielded an additional 13 studies. After applying inclusion criteria, a total of six studies remained (Fig.1). Five of these were randomized controlled trials^7^–^10^,^18^ while the other one was a retrospective cohort trial.^17^ Data extracted from these studies can be found in Table-I. The six included studies contained data detailing 657 (345F, 311M) patients. A total of 336 (127F, 153M) patients received standard surgical dressing treatment, while the other 321 (118F, 158M) patients received NPT. Two studies did not define the gender distribution of their patient sample.^10^,^17^

**Table-I:** Relevant information from included studies.

Study	Country	Study type	Sample size	Age (M ± S.D years)	Superficial surgical site infections	Deep surgical site infections	Seroma formation	Hematoma formation	Re-admission due to complications
O’Neill and Martin (2020)	USA	Randomized controlled trials	SSD: 20 NPT: 20	SSD: 61.2 NPT: 59.6	SSD: 2 NPT: 1	-	-	-	-
Andrianello et al. (2020)	Italy	Randomized controlled trials	SSD: 49 (19F, 30M) NPT: 46 (19F, 27M)	SSD: 64 ± 17 NPT: 69 ± 12	SSD: 3 NPT: 4	SSD: 3 NPT: 1	SSD: 6 NPT: 0	SSD: 1 NPT: 2	-
Javed et al. (2019)	USA	Randomized controlled trials	SSD: 61 (27F, 34M) NPT: 62 (31F, 31M)	SSD: 66.1 ± 9 NPT: 66.4 ± 9.3	SSD: 17 NPT: 4	SSD: 2 NPT: 2	-	-	SSD: 12 NPT: 5
Kuncewitch et al. (2019)	USA	Randomized controlled trials	SSD: 37 (17F, 20M) NPT: 36 (13F, 23M)	SSD: 65 NPT: 65.5	SSD: 6 NPT: 5	SSD: 2 NPT: 3	SSD: 6 NPT: 4	-	SSD: 6 NPT: 3
Shen et al. (2017)	USA	Randomized controlled trials	SSD: 133 (64F, 69M) NPT: 132 (55F, 77M)	SSD: 62 NPT: 59.5	SSD: 17 NPT: 17	SSD: 4 NPT: 4	SSD: 8 NPT: 7	SSD: 0 NPT: 1	SSD: 6 (119) NPT: 3 (118)
Gupta et al. (2017)	USA	Retrospective cohort study	SSD: 36 NPT: 25	-	SSD: 2 NPT: 0	SSD: 9 NPT: 1	-	-	-

Legends: M: Mean: S.D: Standard deviation, F: Female, M: Male, SSD: Standard surgical dressing, NPT: Negative pressure wound therapy.

Average patient age was 63.8 ± 3.15 years. The average age of patients receiving standard surgical dressings was 63.6 ± 2.0 years, while the average age of patients receiving NPT was 64 ± 4.2 years. One study did not report the age of their patient sample.^17^

### Quality assessment for included randomized controlled trials:

**Supplementary Fig.1:**
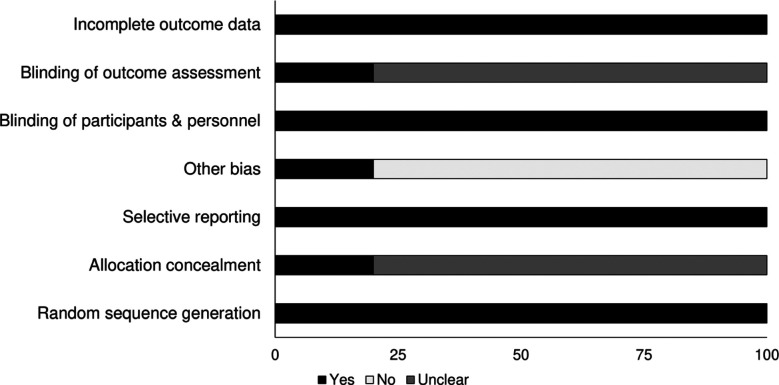
Risk of bias according to Cochrane’s risk of bias assessment tool for randomized controlled trials.

Risk of methodological bias in RCTs was evaluated using Cochrane’s risk of bias assessment tool for randomized controlled trials (Supplementary Table-I). Overall risk of bias was found to be low in the included studies. We observed that allocation of concealment, blinding of participants, and other biases were the most common areas of bias (Supplementary Fig.1).

**Supplementary Table-I:** Demonstrates the risk of bias according to Cochrane’s risk of bias assessment tool for randomized controlled trials (+: low risk, -: high risk, ?: unclear).

Study	Random sequence generation	Allocation concealment	Selective reporting	Other bias	Blinding of participants & personnel	Blinding of outcome assessment	Incomplete outcome data
O’Neill and Martin (2020)	+	?	+	-	+	?	+
Andrianello et al. (2020)	+	?	+	-	+	?	+
Javed et al. (2019)	+	?	+	-	+	?	+
Kuncewitch et al. (2019)	+	?	+	-	+	?	+
Shen et al. (2017)	+	+	+	+	+	+	+

### Quality assessment for included cohort studies:

Risk of methodological bias in retrospective cohort studies were assessed with the ROBINS-I tool (Supplementary Table-II) The overall risk was found to be high in the one included cohort study. We observed that missing data, selection bias, and selective reporting of results were the main biased areas.

**Supplementary Table-II:** Demonstrates the risk of bias according to ROBINS-I tool (+: low risk, -: high risk, ?: unclear)

Study	Confounding bias	Selection bias	Deviation from intended intervention	Missing data	Measurement in outcome	Selection of reported result	Classification of intervention
Gupta et al. (2017)	+	?	+	-	+	?	+

### Publication bias:

**Supplementary Fig.2:**
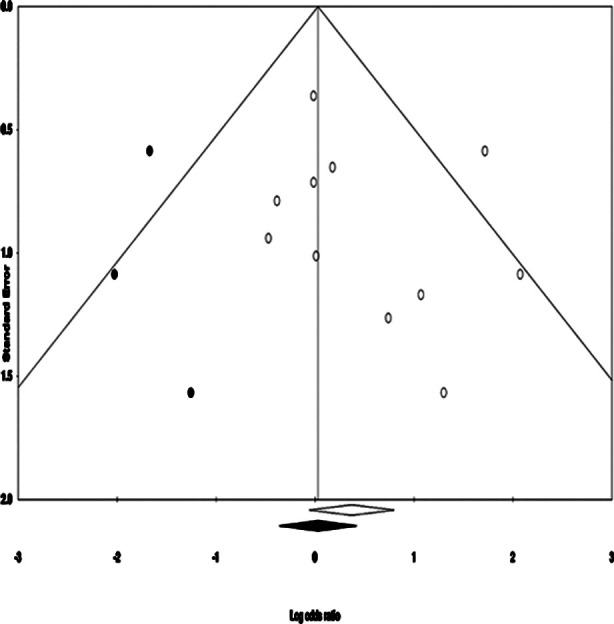
Demonstrates the publication bias by Duval & Tweedy’s trim and fill method.

We used Duval and Tweedy’s trim and fill method to determine missing studies according to the random effects model on either side of the mean effect of the funnel plot (Supplementary Fig.2). Three studies were missing on the left side of the mean effect. The overall random effects model determined the point estimates and the 95% confidence intervals for all the combined studies as 1.50 (0.91 to 2.47). After using the trim and fill, the imputed point estimates were estimated as 1.05 (0.56 to 1.97).

### Meta-analysis report

### Superficial surgical site infection incidence:

**Fig.2:**
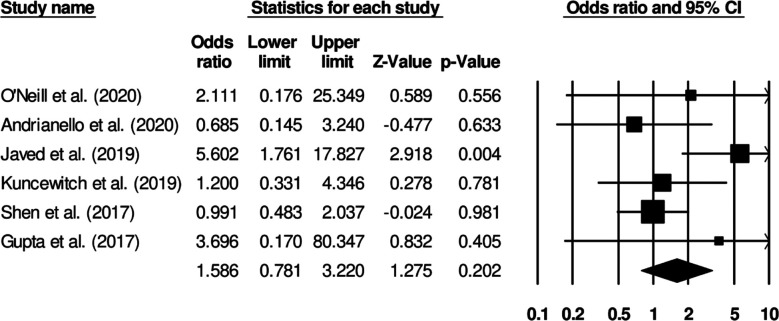
Forest plot for studies evaluating the odds of superficial surgical infection in patients receiving a standard surgical dressing or negative pressure wound therapy.

Six studies reported on superficial surgical infection incidence in patients receiving standard surgical dressing or negative pressure wound therapy. Patients receiving standard surgical dressings presented increased odds of developing superficial surgical infections (Fig.2) (Odds ratio: 1.58, 95% C.I: 0.78 to 3.22, p=0.20). No study heterogeneity was noted (I^2^: 0%).

### Deep surgical site infection:

**Fig.3:**
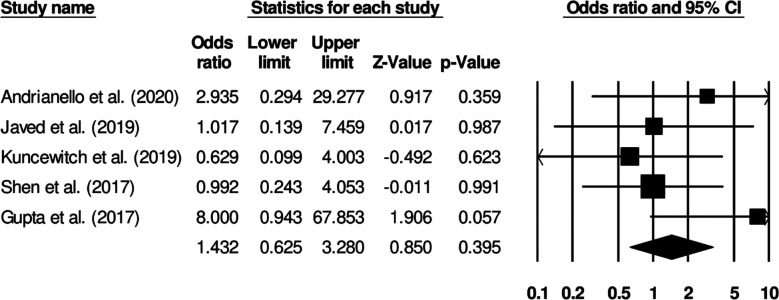
Forest plot for studies evaluating the odds of deep surgical infection in patients receiving a standard surgical dressing or negative pressure wound therapy.

Five studies reported deep surgical infection incidence in patients receiving standard surgical dressing or negative pressure wound therapy. Patients receiving standard surgical dressing presented increased odds of deep surgical infection (Fig.3) (Odds ratio: 1.43, 95% C.I: 0.62 to 3.28, p=0.39). No study heterogeneity was noted (I^2^: 0%).

### Seroma formation:

**Fig.4:**
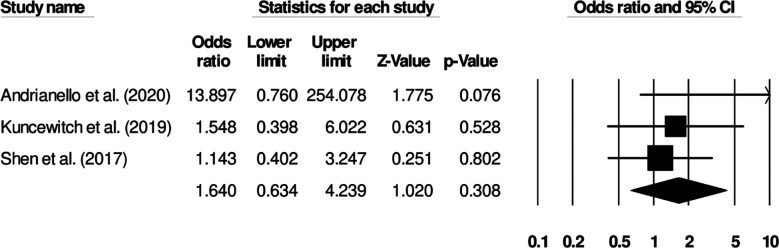
Forest plot for studies evaluating the odds of seroma in patients receiving a standard surgical dressing or negative pressure wound therapy.

Three studies investigated seroma incidence in patients receiving standard surgical dressing or negative pressure wound therapy. Patients receiving standard surgical dressing showed increased odds of seroma incidence (Fig.4) (Odds ratio: 1.64, 95% C.I: 0.63 to 4.23, p=0.30). Study heterogeneity was negligible (I^2^: 10.6%).

### Hematoma formation:

**Fig.5:**
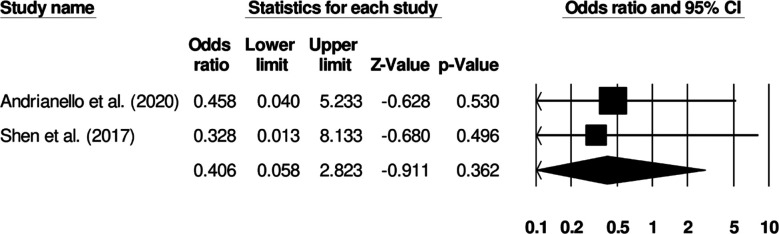
Forest plot for studies evaluating the odds of hematoma in patients receiving a standard surgical dressing or negative pressure wound therapy.

Two studies reported hematoma incidence in patients receiving standard surgical dressing or negative pressure wound therapy. Patients receiving negative pressure wound therapy showed increased odds of hematoma incidence (Fig.5) (Odds ratio: 0.40, 95% C.I: 0.05 to 2.82, p=0.36). No study heterogeneity was noted (I^2^: 0%).

### Hospital re-admission:

**Fig.6:**
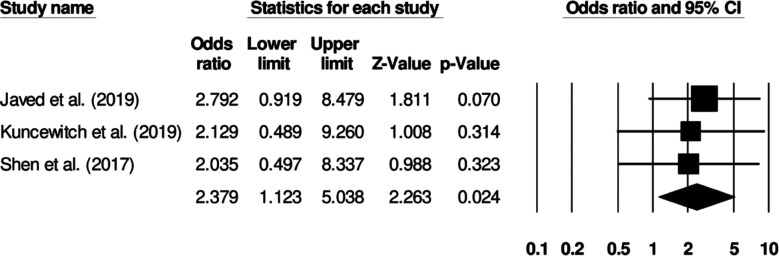
Forest plot for studies evaluating the odds of hospital re-admission in patients receiving a standard surgical dressing or negative pressure wound therapy.

Three studies investigated hospital re-admission rates in patients receiving standard surgical dressing or negative pressure wound therapy Patients receiving standard surgical dressing showed increased odds of hospital re-admission (Fig.6) (Odds ratio: 2.37, 95% C.I: 1.12 to 5.03, p=0.024). No study heterogeneity was noted (I^2^: 0%).

## DISCUSSION

This systematic review and meta-analysis shows increased risk of superficial and deep surgical site infection in patients receiving standard surgical dressings after hepatopancreatobiliary surgery compared to those receiving NPT. We also find elevated risk of seroma and hospital re-admission for patients receiving standard surgical dressings, but an elevated risk for hematoma in patients receiving NPT.

Surgical site infection management is challenging, and patients with surgical site infections after hepatopancreatobiliary surgery exhibit poorer morbidity and mortality-related outcomes.^1^,^2^,^4^ Many researchers have recommended the administration of specialized NPT to avoid or ameliorate these adverse outcomes,^26^,^27^ suggesting that NPT can not only improve morbidity outcomes, but also result in better patient adherence to treatment, treatment cost-effectiveness, and overall quality of life.^28^,^29^ However, while the consensus indicates NPT’s superiority over standard medical dressing for managing normal wounds, no consensus regarding NPT’s efficacy after hepatopancreatobiliary surgery currently exists.

Here, although there was a collective trend towards decreased risk in patients receiving NPT, not all of the individual studies concurred. While Andrianello et al.^7^ reported higher superficial surgical site infection incidence in patients receiving NPT, others Javed et al. 2019, O’Neill and Martin, 2020, Gupta et al.^8^,^10^,^17^ showed the opposite. Andrianello et al.^7^ also found increased rates of organ space infection and post-pancreatectomy hemorrhage in the NPT group. Cohort variation (i.e. the presence of higher body mass index values and more co-morbidities) may account for this discrepancy by affecting surgical procedure and wound management complexity.

Similarly, a lack of consensus also existed for deep surgical site infections between examined studies. Shen et al.^18^ reported no differences in the rate of deep surgical site infection between wound treatment groups. However, Gupta et al.^17^ reported the opposite. The actual nature of the surgical procedure being examined may be the cause of this discrepancy. While NPT can diminish wound surface inflammation, a sealed incision may prevent NPT’s ability to remove excess fluid. Nonetheless, collectively, the risks of both superficial surgical site infection and deep surgical site infections were higher in patients receiving standard surgical dressing as compared to NPT after hepatopancreatobiliary surgery. Furthermore, NPT also directly influences healthcare costs^8^,^17^ by reducing hospital re-admission—something that the present meta-analysis supports.

The present meta-analysis also attempted to reach a consensus concerning the impact of wound treatment approach on seroma and hematoma incidence. Andrianello et al. (2020)^7^ noted that while NPT was successful in preventing seroma onset, it was not successful in limiting hematoma-based complications. Shen et al.^18^ also reported a similar pattern. It is possible that reduced seroma incidence in NPT-receiving patients results from enhanced lymphatic circulation induced by NPT.^30^,^31^ However, it is unclear why NPT is ineffective in limiting hematoma.

### Limitations of the study:

First and foremost, this study was not pre-registered in a systematic review repository such as PROSPERO York or Joanna Briggs Institute owing to logistical issues raised by the current COVID-19 pandemic crisis. Second, we acknowledge the relative scarcity of available data may bias our understanding of the comparative impact of NPT and standard surgical dressing on hematoma complications. As only two studies (featuring small sample sizes) investigated this outcome, incurring a type II error cannot be ruled out.^32^ We therefore strongly recommend future studies to address these limitations by improving the amount of data available on this subject.

## CONCLUSION

This meta-analysis provides preliminary evidence showing that NPT is superior to standard surgical dressing practices for reducing the risks of hospital readmission in patients undergoing hepatopancreatobiliary surgery. We did not observe any significant effect of NPT as compared to standard surgical dressing on superficial, deep surgical infections, seroma, and haematoma outcomes following hepatopancreatobiliary surgery. The findings from the present study cautiously recommend the administration of NPT for managing wound recovery in patients undergoing hepatopancreatobiliary surgery.

### Authors’ contributions:

**BR** conceived and designed the study.

**XJ, JC and JM** collected the data and performed the analysis.

**BR** was involved in the writing of the manuscript and the integrity of the study.

All authors have read and approved the final manuscript.
